# Stunting and academic trajectory in urban settings of Burkina Faso

**DOI:** 10.1371/journal.pone.0314051

**Published:** 2024-12-11

**Authors:** Rabi Joël Gansaonré, Lynne Moore, Jean-François Kobiané, Aly Sié, Slim Haddad

**Affiliations:** 1 Faculté de Médecine, Département de Médecine Sociale et Préventive, Université Laval, Québec, QC, Canada; 2 VITAM–Centre de Recherche en Santé Durable de l’Université Laval, Québec, QC, Canada; 3 Axe Santé des Populations et Pratiques Optimales en Santé, Traumatologie–Urgence-Soins intensifs, Centre de Recherche du CHU de Québec ‐ Université Laval (Hôpital de l’Enfant-Jésus), Université Laval, Québec, QC, Canada; 4 Institut Supérieur des Sciences de la Population, Université Joseph Ki-Zerbo, Ouagadougou, Burkina Faso; 5 Centre de Recherche en Santé de Nouna, Nouna, Burkina Faso; 6 Direction de la Santé Publique, Centre Intégré Universitaire de Santé et de Services Sociaux de la Capitale-Nationale, Québec, QC, Canada; National Research Centre, EGYPT

## Abstract

**Background:**

Impaired growth in childhood can lead to poor cognitive development and low school performance. However, literature on the effects of stunting on school trajectory is very limited. The primary objective of this research was to estimate the age at which children start school according to levels of height-for-age z-score (stunting). A second objective was to estimate the gain in terms of age at school entry associated with an improvement in height-for-age z-score. A third objective was to explore the relationship between stunting, grade repetition, and school dropout.

**Methods:**

We used longitudinal data from the Ouagadougou (Burkina Faso) Health and Demographic Surveillance System. Data from a 2010 health survey of children under 5 years of age were merged with subsequent longitudinal schooling data. The study included 767 children globally who participated in the health and education surveys. Education data allowed us to apprehend academic trajectory measured by age at school entry, repetition, and dropout. The health survey gathered anthropometric information that was used to measure stunting. The adjusted age at school entry was estimated using a Poisson model. The gain represents the difference in adjusted age at school entry for different values of height-for-age. The relationship between stunting and grade repetition and dropout was studied using a discrete-time survival model.

**Results:**

Results showed that children entered school on average at 5.7 years old, and the incidence of grade repetition and dropout was 17.7 and 6.6 per 100 persons-years, respectively. The adjusted age at school entry of severely stunted children was 6.2 years [95% confidence intervals (CI): 6.1; 6.3] compared to 5.1 years [95% CI: 5.0; 5.3] for children who had normal growth. The difference (gain) in adjusted age at school entry between severely and non-stunted children was thus 1.06 [95% CI: 0.87; 1.25] years. If a child’s growth changed from severe stunting to normal growth, their risk of repeating a grade decreased by 5.0 [95% CI: 0.0; 9.0] per 100 persons-years. We did not observe a relationship between height-for-age and dropout.

**Conclusion:**

The results show that schooling is affected in several ways for children who are stunted. The age at school entry of stunted children is more likely to be delayed. Also, being stunted is associated with higher incidence of grade repetition. However, the relationship between stunting and dropout was inconclusive.

## Introduction

In developing countries, more than 200 million children under 5 years of age fail to reach their optimal cognitive development because of poverty and poor health and nutrition [1]. Infections and poor nutrition during childhood can lead to growth impairment [1–3], resulting in short adult height and impaired cognitive development [4]. Also, many children of elementary-school age in developing countries are not enrolled in school, and about one-quarter of those who are enrolled do not complete elementary education [1].

Previous studies show that children who experience growth retardation during childhood are less likely to be enrolled in school [5] or more likely to delay their school enrollment than those who have not experienced growth retardation [6,7]. The literature also shows that impaired growth is associated with lower performance in mathematics and reading, and higher risk of grade repetition and dropout [8,9]. However, the possible effects of growth retardation on academic performance, repetition, and dropout are not fully established. Results are sometimes inconsistent and even contradictory. For example, Yamauchi [10] found that height-for-age z-score was positively associated with the number of completed grades, but the relationship was reversed for high height-for-age z-scores. Other studies did not find an association between height-for-age z-score and grade repetition [10,11], stunting (height-for-age < -2) or height-for-age and school performance [12,13], and height-for-age and completed grade level [13].

Mechanisms by which stunting could lead to difficulties at school are not well known. The literature suggests, however, that the occurrence of stunting in the first years of life could negatively affect brain development and cause cognitive impairment; this relationship could be one link between stunting and school outcomes [1]. Children who experienced stunting could then have delayed motor and language development and poor performance in mathematics and reading [8,14,15]. This situation could then negatively impact children’s school trajectory.

Previous studies have mainly focused on analyzing the association between height-for-age or stunting and educational outcomes, but components of the school trajectory (age at school entry, repetition, and dropout) that determine the level of education are little studied. To our knowledge, no study has evaluated the age at which children start school or the level of grade repetition and school dropout according to height-for-age z-scores. The gain in terms of age at school entry, repetition, and dropout associated with an increase of the height-for-age z-score is unknown. Therefore, it would be helpful to understand how stunted growth influences each component of the school trajectory. This study, thus, fills a gap in the literature on the benefits of better growth in childhood in relation to school trajectory.

The primary objective of this research was to estimate the age at which children start school according to levels of height-for-age z-score (stunting). A second objective was to estimate the gain in terms of age at school entry that would be associated with an improvement in child height-for-age z-score. Our third objective was to explore the relationship between stunting, grade repetition, and school dropout.

## Methods

### Study design and setting

To assess the relationship between child health and schooling, we used longitudinal data collected from children living in the five city districts covered by the Ouagadougou Health and Demographic Surveillance System (OHDSS). Cross-sectional health data collected in 2010, when children were under 5 years old, were combined with their subsequent longitudinal schooling data. In Ouagadougou, the capital of Burkina Faso, many children do not have the opportunity to go to school, and the education system is marked by high levels of repetition and dropout [16,17], which limit the possibility for some children to complete even the compulsory cycle. In 2018, for example, only 83.2% of children aged 6 to 11 were enrolled in elementary school [18]. In the same year, the elementary school completion rate (i.e., the ratio of the number of newly admitted [non-repeaters] in the last grade of elementary school to the 11-year-old population) was 78.5% [18]. Furthermore, many children experienced developmental problems in early childhood. Indeed, the prevalence of stunting was 27.3% among children under 5 years of age in 2016 [19]. The OHDSS provides a useful setting to study the relationship between stunting and school trajectory. The study is reported according to the Strengthening the Reporting of Observational Studies in Epidemiology (STROBE) guideline (see S1 Checklist).

### Study population

Our sample consisted of children who participated in the health survey and follow-up to collect educational data. The education survey principally concerned residents of the OHDSS aged 5 and over. However, children who started school earlier (before age 5) are included in the education follow-up. The health survey concerned 1,941 households. Households were selected randomly to ensure the representativeness of the OHDSS area [20]. Of 1,941 households, 1,699 participated in the survey—a response rate of 87.5% [21]. The health survey gathered data on 950 children under 5 years old. Of them, 767 were enrolled in school and followed from the 2013–14 academic year to 2017–18. Due to death and emigration, follow-up with the remaining 182 children was not possible. Three samples were drawn based on the 767 children (S1 Fig). The first sample consisted of 668 children and allowed us to examine the relationship between height-for-age z-score and age at school entry (S1 Fig). For some students (99, representing 12.9% of 767 children), the age at school entry was unknown because they emigrated, did not enter school, or started school before the beginning of the education survey (see S1 Fig). The second sample included 648 children who attended school for at least two years. This sample was used to test the hypothesis that an increase in child height-for-age z-score is associated with a decrease in the risk of grade repetition (S1 Fig). The association between height-for-age z-score and dropout was assessed based on a third sample of 685 children who have already attended school (some children are still in school; others are no longer in school) (S1 Fig).

### Data sources

As already noted, the data used in the present study are from the OHDSS. The area of the OHDSS consists of five neighborhoods located in the northern part of Ouagadougou [21]. In 2012, 86,400 individuals were followed by the OHDSS; 40,700 of them lived in two formal neighborhoods, and 45,700 lived in three informal neighborhoods. Informal neighborhoods are devoid of formal zoning plans [21]. They are spontaneous settlements without basic public services (school, health center, electricity, and water), and most of the houses are built of clay.

The OHDSS regularly collects data on vital events (births, deaths, unions, migrations) [21]. Data are also collected on education, and, in 2010, a health survey was conducted in the OHDSS area. The present study focuses on those two data sources: education and health data. The health survey was undertaken between February and August 2010 on a random sample of 1,699 households [21]. The education survey gathered data on school and class attendance for 5 school years (2013–14 to 2017–18).

### Outcome measures

The primary outcome of our study was age at school entry, defined as the age at which a child starts to go to school for the first time. Secondary outcomes were grade repetition and school dropout. Information on children’s school status (goes to school, already gone to school, has never been to school) and grades attended allowed us to create three dependent variables that describe school trajectories. School entry age corresponded to the age at which the child’s school status “goes to school” was observed for first time. Repetition is obtained by comparing the successive grades of children, so there is grade repetition if a child attends the same grade for two successive school years. We considered that dropout had occurred if a child was enrolled in school the previous year but no longer attended school during the current school year. In this case, the child’s school status was “already gone to school.” Grade repetition and dropout are right censored for those who do not experience the event at the end of the follow-up.

### Main independent variable

As in the literature, stunting was approached through the height-for-age z-score (standardized height-for-age ratio). Height-for-age z-score can be defined as the deviation of an individual’s height value from the median height value of a reference population [22]. Children’s height-for-age z-score were calculated based on three parameters: height, sex, and age. We used the function *zanthro* of the statistical software STATA [23] to transform child’s height to z-score using the World Health Organization’s child growth standards published in 2006 [24]. Anthropometric data were measured twice by experienced surveyors in order to reduce measurement errors and maximize the reliability of the exposure. Surveyors were trained to collect anthropometric data, and had several previous data collection experiences with the studied population. Age and sex variables were part of the OHDSS, so there are no reliability issues.

### Potential confounders or modifiers

Potential confounders were variables that may be associated with both stunting and school trajectory, including child’s sex, mother’s education level, household socioeconomic status, and place of residence (formal or informal area). Socioeconomic status is a composite variable whose calculation is described elsewhere [25]. Briefly, the indicator of socioeconomic status was constructed from the possession of goods (television and refrigerator) and the household’s main mean of transportation. Principal component analysis was used to determine the coefficient of each variable. Based on the first factor (which explained 50.4% of the total variance), households were classified in three categories (poor, middle, and rich). Households that did not own any assets, neither a refrigerator nor a television, and who reported that they mainly moved by foot or by bicycle were considered “poor” [25]. In the group of households considered to have a “middle” status, about half had a television, and most of them used motorbike as their means of transportation [25]. The “rich” class of households mostly consisted of those who owned a refrigerator or a television [25]. About half of these households had at least one motorbike as means of transportation [25]. Because of a low proportion of rich households, the variable was then recoded into two categories (poor, less-poor) by grouping middle and rich categories into “less-poor.”

Mother’s education was determined based on the information collected about the highest grade residents had completed or the grade they were currently attending. This variable was initially recoded in three categories (no education, primary education, and secondary/higher education). However, due to the low number of mothers who had secondary/higher education, we recoded the mother’s education variable as two categories (no education or some education) by grouping primary education and secondary/higher education into “some education.”

Year of birth was also considered as a confounder. The birth month (January–August or September–December) was added in the analysis of the age at school entry because we assumed that children born earlier in the year were more likely to be enrolled in school in a given year than those born later. Birthweight was identified as a potential confounder, but was not available and therefore not included in the analysis.

### Statistical analysis

Poisson regression with robust standard errors was used to examine the relationship between height-for-age z-score and age at school entry. The linearity assumption not being respected, we included the height-for-age z-score as a quadratic and cubic term. We then calculated the adjusted age at school entry based on the thresholds commonly used to define severe stunting (height-for-age < -3), moderate stunting (height-for-age < -2), mild stunting (height-for-age < -1), and non-stunting/normal growth (height-for-age > = 0) [24,26], using the STATA 18 postestimation command (margins). We assumed that age at school entry would be the same at each point of height-for-age if there is no effect of height-for-age z-score. This hypothesis of change in age at school entry was tested by calculating the estimated gain for different initial values of height-for-age z-score. The gain represents the difference in adjusted age at school entry for different values of height-for-age. We also estimated the gain in age at school entry for each point of height-for-age z-score based on a reference value of the height-for-age z-score. To assess the potential modification effect of sex, socioeconomic status, mother’s education, and place of residence, interaction terms between these variables and height-for-age z-score were included in the model.

A discrete-time survival model allowed us to estimate the probability that repetition or dropout occurred, conditional on the individual still being at risk. The population at risk consisted of children enrolled in school and who had not experienced the event under study. A time variable was included in the model. This model was fitted through logistic regression [27,28]. Ninety-five percent (95%) confidence intervals of hazard rates were estimated using robust standard errors. As for the age at school entry model, adjusted incidence rates were calculated.

### Sensitivity analysis

Due to the absence of the birthweight variable as a covariate, which could lead to a residual confounding of the association between height-for-age and age at school entry, a sensitivity analysis was performed using two instrumental variables: score of household hygiene and score of food security. As in previous studies [2,29], variables use to measure household hygiene are related to sanitation, source of water, household cleanliness, and nature of the floor. In this study, scores of household hygiene were derived from four variables (see S1 Table). Scores were obtained by summing the coefficients associated with each modality of the variables included in the score construction. The hygiene score ranged from 1 (worst hygiene) to 8 (best hygiene). Household food security scale was measure using nine questions (see S2 Table) developed by Coates et al. [30]. The reference period of the questions was the 30 last days prior the survey date. The Coates et al. scale is commonly used to measure food security in developing countries [31]. The household food security scale was calculated using the same method as for household hygiene. The score ranged from 9 (food security) to 36 (food insecurity).

### Ethics approval

The health survey was conducted in 2010. The educational data were gathered between the 2013–14 and 2017–18 academic years. The OHDSS has obtained ethical approval from the Comité National d’Éthique pour la Recherche en Santé for the collection of health and routine data, including educational information. The approval references for the collection of health and routine data are 2010–010 and 2010–009, respectively. In addition, written informed consent was obtained from household heads. The written informed consent was signed by the head of household who agreed to their household’s participation in the study. Information on the children was provided by their mothers or caregivers. The data provided to the authors was completely anonymous.

## Results

Table 1 presents descriptive statistics of participants. Globally, children entered school at 5.7 years old. The incidence of grade repetition was 17.7 per 100 persons-years over the period of study, while the incidence of dropout was 6.5 per 100 persons-years. In sample 1, the mean height-for-age was -1.64, suggesting that children in our sample are generally shorter than the reference population. Our main sample (sample 1) was predominantly male (53.0%). The majority was from less disadvantaged families (56.2%), lived in an informal area (62.4%), and their mother was not educated (Table 1).

**Table 1:** Age at school entry, incidence of grade repetition, incidence of dropout, and baseline characteristics of participants.

Variables	Sample 1 Age at school entry		Sample 2 Grade repetition		Sample 3 Dropout	
	N (668)	%^§^	N (648)	%^§^	N (685)	%^§^
Age at school entry ^ƿ^	668	5.67 (3;9)	-	-	-	-
Grade repetition^¶^	-	-	1723	17.70	-	-
Dropout^¶^	-	-	-	-	2498	6.57
Height-for-age z-score^ƿ^	607^μ^	-1.63 (-5.87;5.08)	589^&^	-1.59 (-5.87;5.08)	623^$^	-1.61 (-5.87;5.08)
Child’s sex						
Boy	306	45.8	300	46.3	320	46.7
Girl	362	54.2	348	53.7	365	53.3
Year of birth						
2005	105	15.7	103	15.8	108	15.7
2006	156	23.4	155	23.9	161	23.5
2007	155	23.2	152	23.5	158	23.1
2008	185	27.8	172	26.6	188	27.4
2009	67	10.0	66	10.1	71	10.3
Month of birth						
January–August	431	64.6	-	-	-	-
September–December	237	35.4	-	-	-	-
Household socioeconomic status^β^						
Poor	285	42.7	275	42.4	292	42.6
Less-poor	382	57.2	371	57.3	391	57.1
Missing	1	0.1	2	0.3	2	0.3
Mother’s education^γ^						
No education	406	60.8	390	60.2	416	60.7
Some education	223	33.4	227	35.0	232	33.9
Missing	39	5.8	31	4.8	37	5.4
Place of residence^δ^						
Formal	253	37.9	242	37.4	258	37.7
Informal	414	62.0	404	62.3	425	62.0
Missing	1	0.1	2	0.3	2	0.3
Food security score^†^	660^θ^	18 (13;23)	-	-	-	-
Household hygiene score^†^	660^θ^	6 (5;7)	-	-	-	-

^¶^ The proportion is calculated based on person-time date. It is an incident rate and is interpreted as number of cases per 100 persons-years.

^ƿ^ Mean(min; max).

^†^Median(q1-q3).

^γ^ Mothers with no education were those with no formal education. Those who attended to formal education (elementary education or more) were grouped into “some education.”

^β^ The less-poor households were grouped together with the rich and intermediate households. They were grouped together because of the low number of rich households.

^δ^ Informal area comprised Polesgo, Nonghin, and Nioko II. They are devoid of zoning plan and basic public services (schools, health centers, etc.). Formal neighborhoods (Kilwin and Tanghin) are well structured and provided with public infrastructure.

^μ^ Missing = 61 (9.1%)

^&^ Missing = 59 (9.1%)

^$^ Missing = 62 (9.1%)

^θ^ Missing = 8 (1.2%).

**Fig 1:**
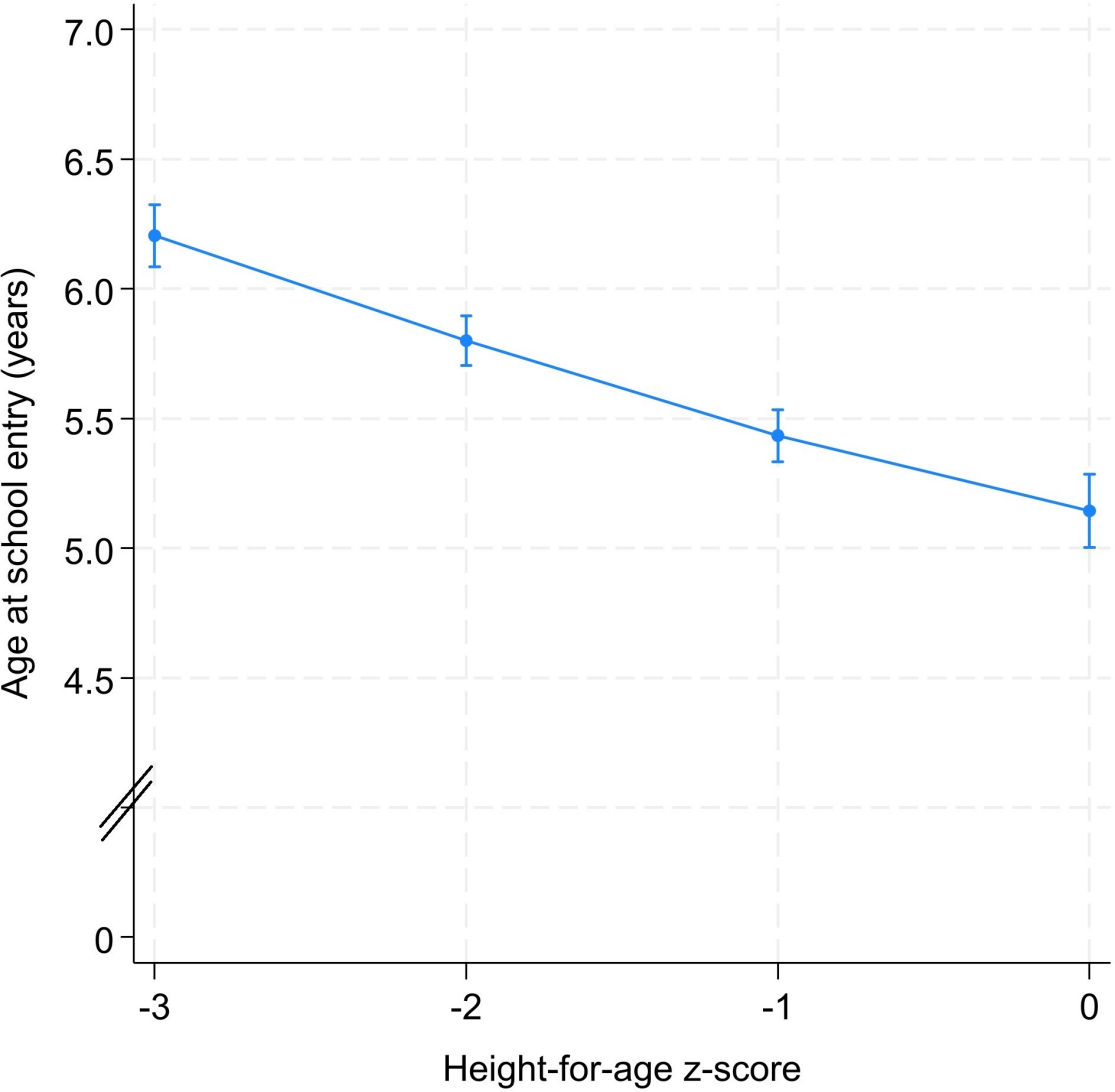
Predicted^*^ value of age at school entry (in years), by level of height-for-age. ^*^Model was adjusted for sex, year of birth, month of birth, household socioeconomic status, mother’s education, place of residence.

An examination of the relationship between height-for-age z-score and age at school entry showed that, globally, an increase of one unit of height-for-age z-score was associated with a decrease in age at school entry (Fig 1). For example, the adjusted mean age at school entry was 6.2 [95% confidence intervals (CI): 6.1; 6.3] years for children with a height-for-age z-score of -3. This age was 5.8 [95% CI: 5.7; 5.9] and 5.4 [95% CI: 5.4; 5.5] years for those with a z-score of -2 and -1, respectively (Fig 1). The age at school entry reached its minimum when the height-for-age z-score was equal to 0.

Table 2 and S2 Fig show the change in age at school entry for each unit increase in height-for-age z-score. The school entry age of a child decreased by 0.40 [95% CI: 0.33; 0.48] if their health status moved from severe to moderate stunting (Table 2 and S2 Fig). The difference (gain) in adjusted age at school entry between severely stunted children and non-stunted children was 1.06 [95% CI: 0.87; 1.25] years (Table 2).

**Table 2:** Change^a^ in age at school entry [95% confidence interval].

Height-for-age	Reference height-for-age		
	-3	-2	-1
-3	0 [;]		
-2	-0.40 [-0.47; -0.33]	0 [;]	
-1	-0.77 [-0.90; -0.64]	-0.37 [-0.44; -0.29]	0 [;]
0	-1.06 [-1.25; -0.87]	-0.66 [-0.80; -0.51]	-0.29 [-0.37; -0.21]

^a^ Changes were calculated based on predicted mean of age at school entry.

Model was adjusted for sex, year of birth, month of birth, household socioeconomic status, mother’s education, place of residence.

**Fig 2:**
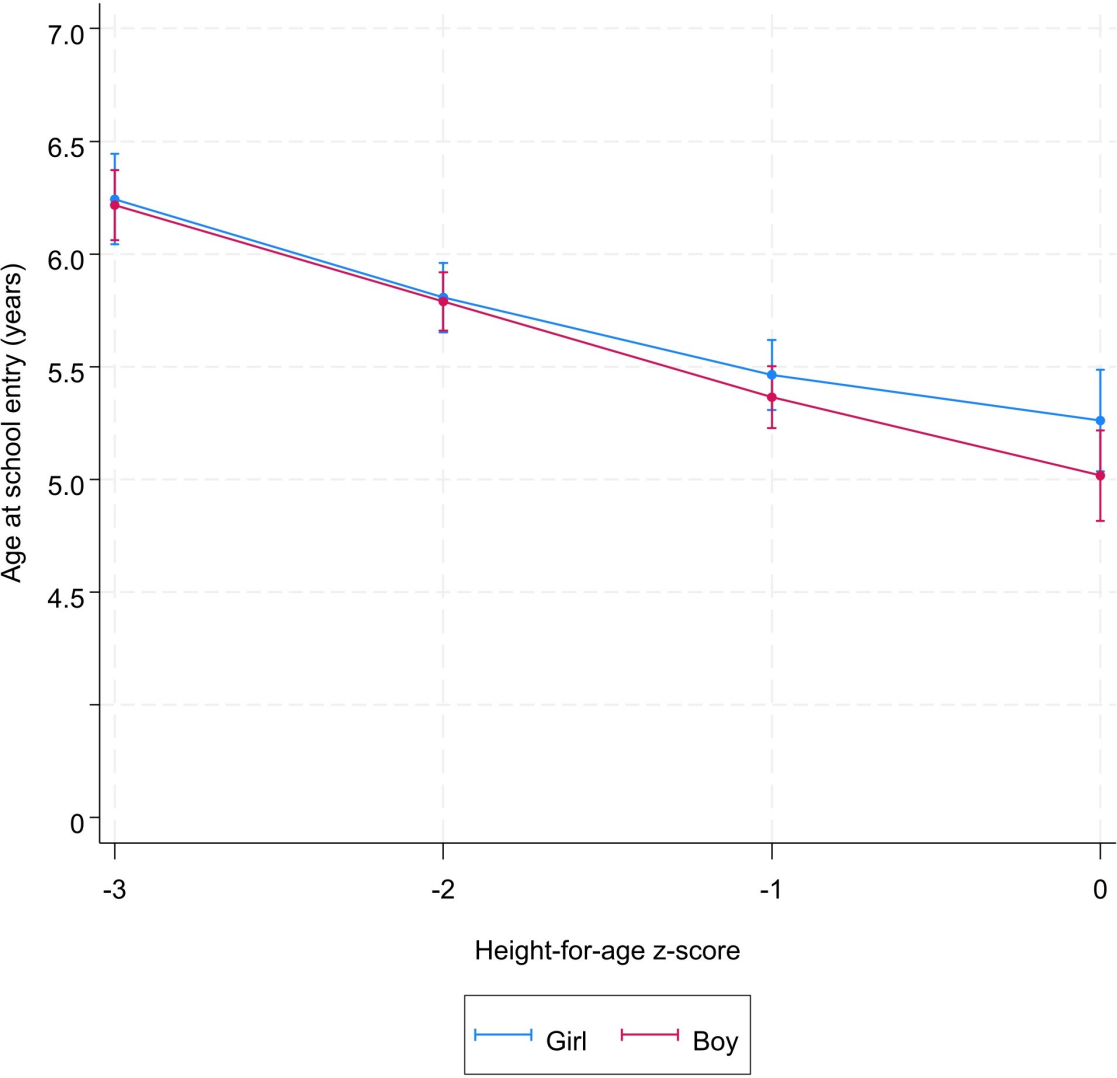
Predicted^*^ value of age at school entry by height-for-age z-score and child’s sex. ^*^Model was adjusted for sex, year of birth, month of birth, household socioeconomic status, mother’s education, place of residence.

**Fig 3:**
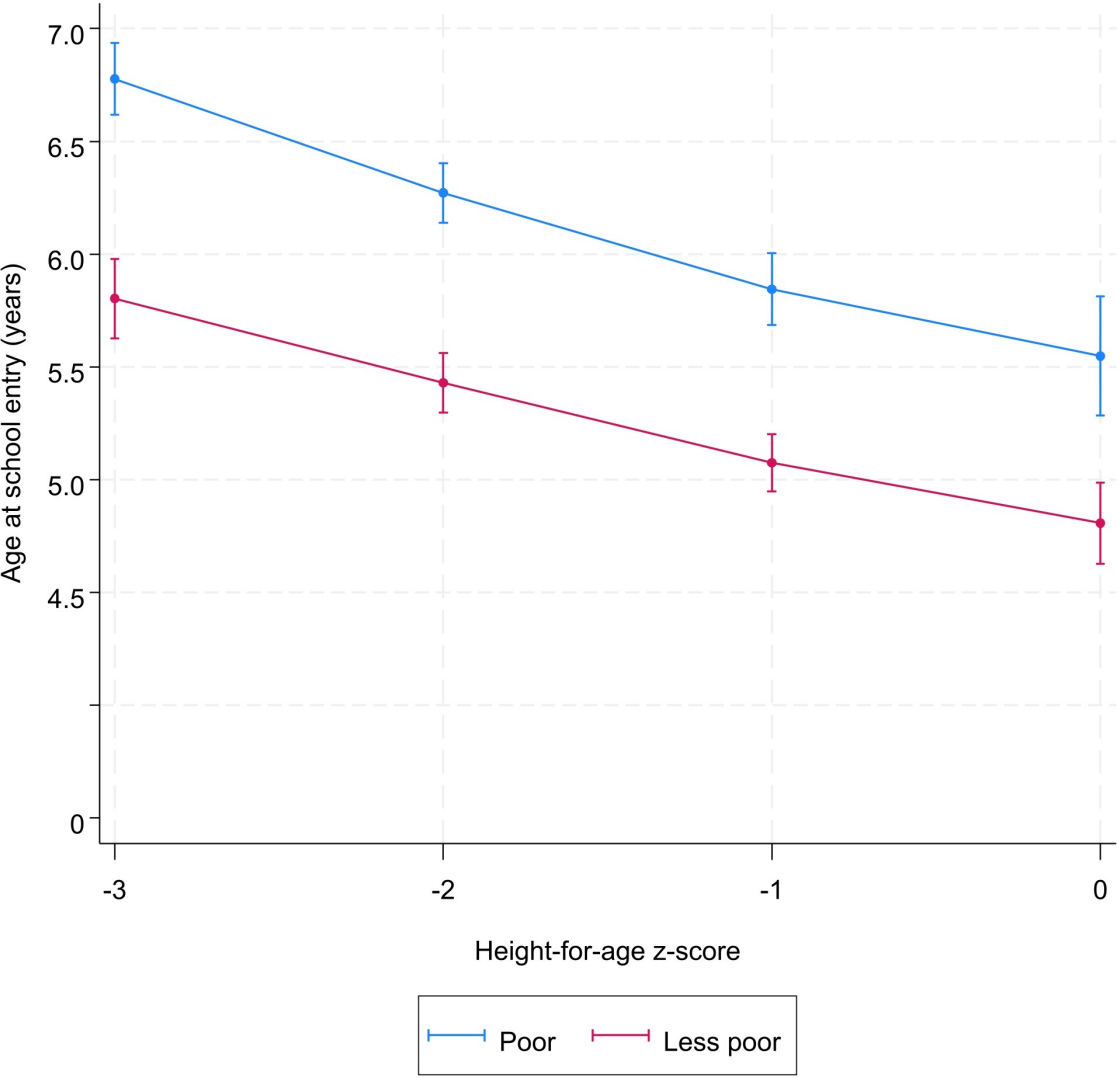
Predicted^*^ value of age at school entry by height-for-age z-score and household socioeconomic status. ^*^Model was adjusted for sex, year of birth, month of birth, household socioeconomic status, mother’s education, place of residence.

**Fig 4:**
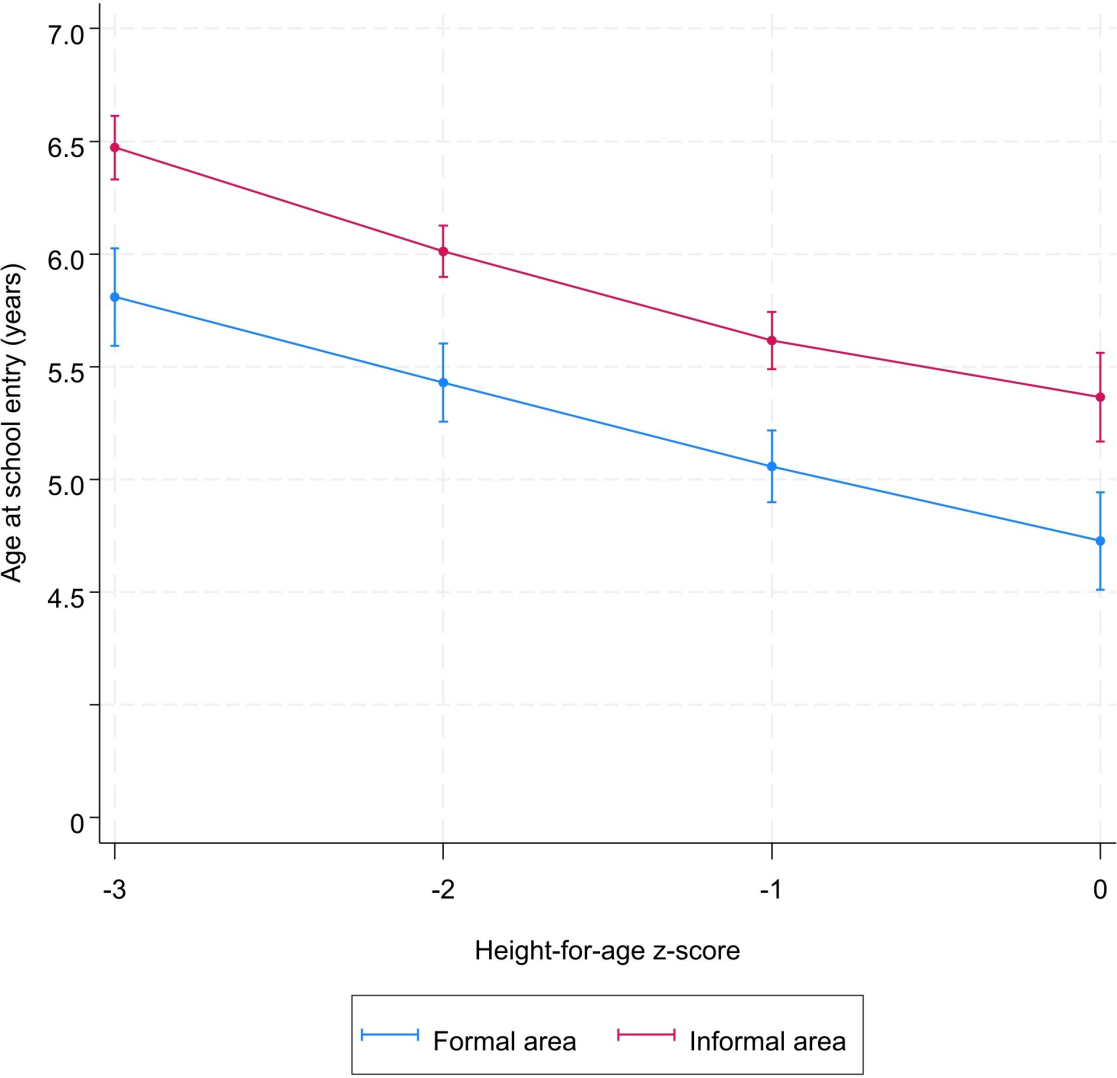
Predicted^*^ value of age at school entry by height-for-age z-score and place of residence. ^*^Model was adjusted for sex, year of birth, month of birth, household socioeconomic status, mother’s education, place of residence.

**Fig 5:**
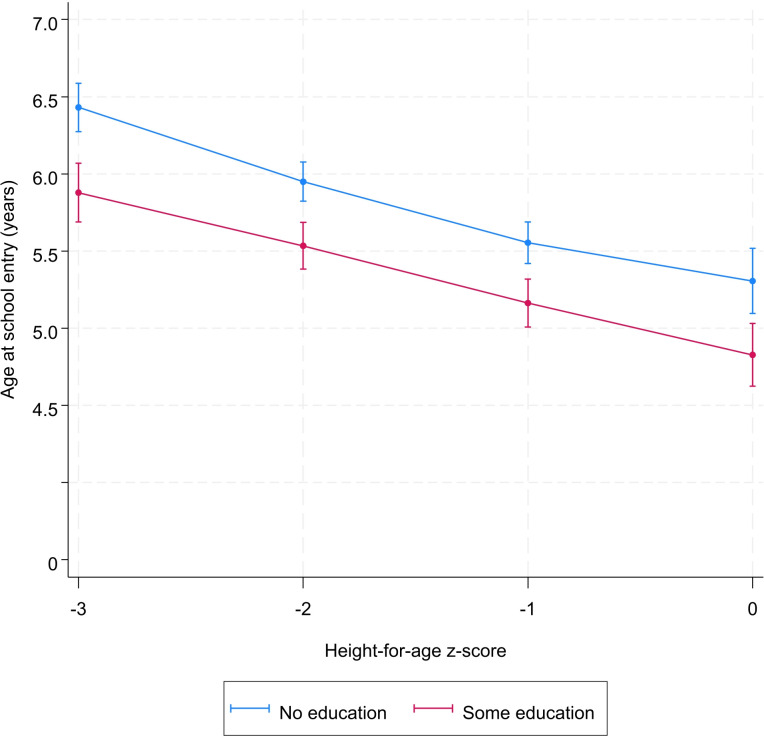
Predicted^*^ value of age at school entry by height-for-age z-score and mother’s education. ^*^Model was adjusted for sex, year of birth, month of birth, household socioeconomic status, mother’s education, place of residence.

Sex, socioeconomic status, place of residence, and mother’s education did not modify the association between height-for-age and school starting age (Figs 2–5 and S3 Table). However, irrespective the value of the height-for-age, the age at school entry of children from poor households, from informal areas, and those whose mother are uneducated was higher than those from less-poor households, formal areas, and those whose mother are educated, respectively (Figs 2–5 and S3 Table).

**Fig 6:**
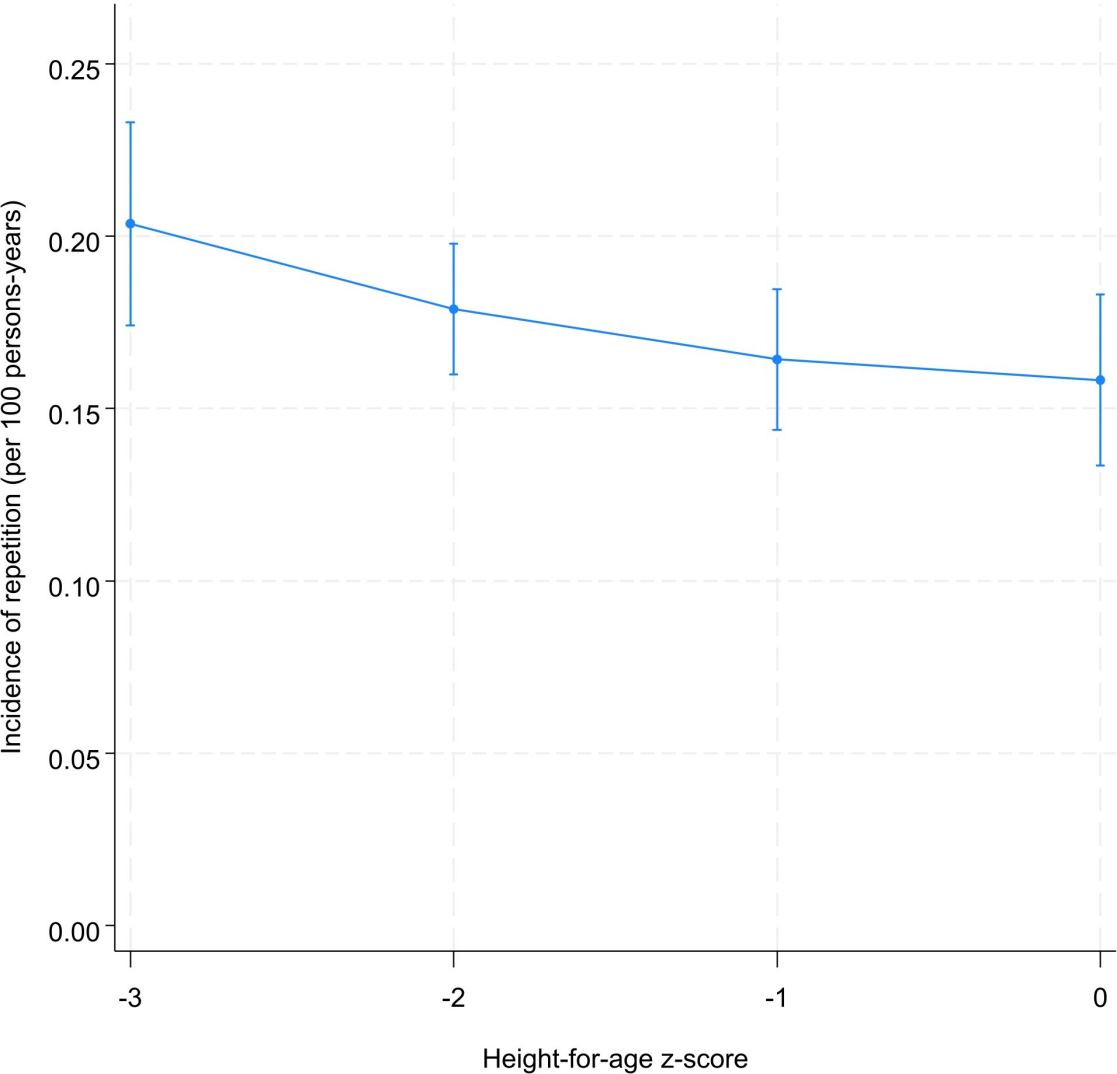
Predicted^*^ incidence of repetition by level of height-for-age. ^*^Model was adjusted for sex, year of birth, month of birth, household socioeconomic status, mother’s education, place of residence.

**Fig 7:**
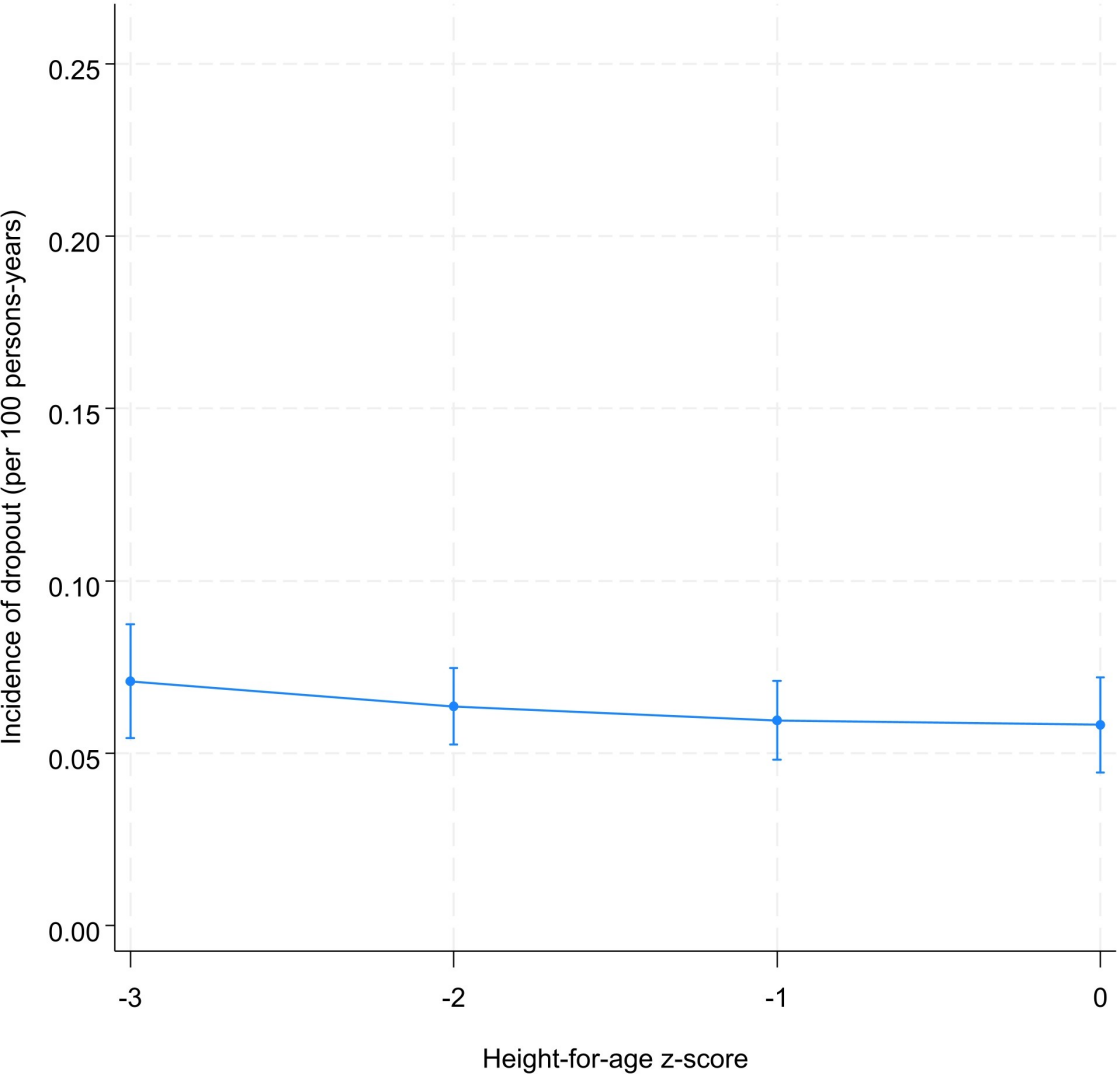
Predicted^*^ incidence of dropout by level of height-for-age. ^*^Model was adjusted for sex, year of birth, month of birth, household socioeconomic status, mother’s education, place of residence.

The pattern of incidence of repetition is comparable to that observed for the age at school entry. The incidence of grade repetition decreased as the health status improved and reached its minimum when the stunting is eliminated (Fig 6). Grade repetition was 20.4 [95% CI: 17.4; 23.3] and 15.8 [95% CI: 13.3; 18.3] per 100 persons-years for severely stunted and non-stunted children, respectively (Fig 6). We were not able to make a definitive conclusion about the association between height-for-age and dropout (Fig 7). However, it seems that there is a gradient between these two variables. Indeed, the incidence of dropout was 7.1, 6.4, and 6.0 per 100 persons-years for severely, moderately, and non-stunted children, respectively (Fig 7).

By testing the difference between incidence rates of repetition at different initial values of height-for-age, we observed a decrease in grade repetition as height-for-age increase from -3 to -2 (-0.02 per 100 persons-years, 95% CI: -0.05; -0.00) and from -2 to -1 (-0.01 per 100 persons-years; 95% CI: -0.03; -0.00) (Table 3 and S3 Fig). For a child with a z-score of -3, the risk of experiencing a first repetition decreased by 5.0 [95% CI: 0.0; 9.0] per 100 persons-years if the z-score moved to 0 (Table 3). The tests of difference between the incidence rates of dropout at different levels of stunting were inconclusive (S4 Fig).

**Table 3:** Change^a^ in incidence of repetition [95% confidence interval].

Height-for-age	Reference height-for-age		
	-3	-2	-1
-3	0 [;]		
-2	-0.02 [-0.05; -0.00]	0 [;]	
-1	-0.04 [-0.07; -0.01]	-0.01 [-0.03; -0.00]	0 [;]
0	-0.05 [-0.09; -0.00]	-0.02 [-0.04; 0.00]	-0.01 [-0.02; 0.01]

^a^ Changes were calculated based on predicted incidence of grade repetition.

Model was adjusted for sex, year of birth, month of birth, household socioeconomic status, mother’s education, place of residence.

### Sensitivity analysis

Sensitivity analysis shows that the height-for-age is exogenous. Indeed, the rho statistic (measure of the strength of the endogeneity) from the adjusted model was almost null [ρ = 0.02 (95% CI: -0.03; 0.06)], suggesting that the instrumental variable model is not necessary (S4 Table). The incident rate ratios from the Poisson model and those from the Poisson model with an endogenous covariate were very similar (see S4 Table).

## Discussion

This study validated our hypothesis that stunting is associated with age at school entry. For children who were severely stunted, the age at school entry was 6.2 years [95% CI: 6.1; 6.3] compared to 5.1 years [95% CI: 5.0; 5.3] for those who had normal growth. The gain associated with an elimination of stunting from a severely stunted children was thus 1.06 years [95% CI: 0.87; 1.25].

Our findings are consistent with previous studies. For example, a study undertaken in Kwazulu-Natal (South Africa) using longitudinal data found that the age at which children started school decreased by 0.148 years for each unit increase of the standardized height-for-age ratio [10]. A study showed that stunted children were less likely to be underage at enrollment and almost twice as likely to be overage at the point of entry compared to children who were not stunted [9]. In Burkina Faso, the legal school entry age is 6 years [32]; thus, non-stunted children in this study were underage when they entered school. This finding is consistent with previous studies. For example, a study on young Filipino children found that an increase in height-for-age z-score was associated with an increase in the probability of earlier enrollment and a decrease in the probability of later enrollment [33]. The school enrollment of children with low height-for-age is probably delayed because their families consider them unready to start school earlier [6]. Also, the age at school entry can be delayed because certain faculties (communication, psychomotor, and mental) are not very well developed [9]. Indeed, it is well demonstrated in the literature that being stunted is associated with poor cognitive development at 5 years of age, poor psychomotor development, and poor mental development [34,35]. A prospective study on Tanzanian children showed that height-for-age was linearly associated with cognitive, communication, and motor development z-score [36].

The age at school enrollment is higher for children whose mothers are uneducated, from poor households, and live in informal areas compared to those whose mothers have some education, come from less disadvantaged households, and live in formal areas, whatever the health status. These findings could be related to poor cognitive development during childhood among children from poor socioeconomic conditions and informal areas, and whose mothers are uneducated. In fact, a study realized in Brazil using longitudinal data in an urban context showed that unfavorable socioeconomic conditions and poorly educated mothers were negatively associated with cognitive performance at 5 years of age [34]. These findings also show the negative role that household poverty, mother’s low level of education, and living in rural or informal area can play in the delay of school entry, as studies on socioeconomic determinants of delayed school entry have also found [37,38].

The incidence of grade repetition decreased for each increase of a unit of height-for-age z-score. If a child’s health moved from severe stunting to normal growth, their risk of repeating a grade decreased from 20.4 [95% CI: 17.4; 23.3] to 15.8 [95% CI: 13.3; 18.3] per 100 persons-years. The association between grade repetition and stunting suggests that special attention should be given to children who have had growth retardation in order to improve their school performance. In line with our study, a recent systematic review in developing countries found that stunted children had higher odds of grade repetition compared to non-stunted children [39]. Our findings are also consistent with a previous study conducted in the Philippines that revealed that one standard deviation increase in height was associated with a reduction of the probability of repeating the first grade [6]. Also, in Malawi, Gandhi et al. [13] show that height gain during early childhood was negatively associated with grade repetition [13]. However, one study in Indonesia was inconclusive regarding the association between stunting and grade repetition [40]. The high level of grade repetition of stunted children or children with negative height-for-age z-scores could lead to lower schooling levels [9]. Indeed, the process by which stunting occurs probably leads to structural and functional brain damage, which in turn causes delayed cognitive development [4,41].

Like Bogin and MacVean [42], we were not able to find an association between stunting and one of our secondary outcomes—dropout. However, results revealed that a gradient seems to be emerging between the two variables. Although the association was not conclusive, the incidence of school dropout tends to decrease when the stunting changes from severe to no stunting. In the literature, the relationship between stunting and school dropout is mitigated. Indeed, using data from the Cebu Longitudinal Health and Nutrition Survey, Mendez and Adair [43] show that stunted children at age 2 were more likely to have dropped out of school by age 11 (OR: 3.0, 95% CI: 1.5; 5.8), compared to those who were not stunted at age 2 [43]. In contrast, a study on Ghanian children aged 6 to 12 years old in 1988–89 shows that being tall for their age (non-stunted) was associated with a higher probability of dropout compared to children who were short for their age [44].

Several hypotheses can be evoked to explain the fact that the analysis of the association between stunting and dropout was inconclusive. First, this study was undertaken in an urban context where a modern health system is available [45] and the population, including children, has access to health centers [21]. Access to health services, therefore, can contribute to reducing the potential effect of growth impairment on children’s school trajectory. Second, the implementation of the mandatory schooling policy for children aged 6 to 16, adopted by the government of Burkina Faso in 2007 [32], may have contributed to keeping children in school and reducing school dropout. Third, it is possible that children who have difficulties at school receive support from their parents in learning (e.g., supervising exercises, providing books and fees, etc.), which could help prevent school dropout [46–48]. A fourth possible reason for the inconclusive association between stunting and school dropout is the limited number of participants in our study. Future studies could be conducted to investigate whether there is a relationship between stunting and school dropout.

This study has some limitations. First, a possible selection bias was introduced in the analysis of age at school entry because we were not able to include 12.9% of eligible participants in that analysis. The exclusion was due to the non measurement of the outcome variable (age at school entry) for these children because they were enrolled at the school before the school follow-up began. However, the tests of independence comparing the observed characteristics (height-for-age, sex, month of birth, year of birth, socioeconomic status, mother’s education, place of residence, food security score, household hygiene score) of children with and without data on age at school entry showed that the two groups had the same distribution, which suggests that missing data may not affect our results (see S5 Table). Second, there is a possible confounding factor—namely, birthweight—that we could not include in the analysis. Birthweight was not measured and therefore could not be considered in the analysis. However, we carried out sensitivity analyses using an instrumental variable model, which did not suggest the presence of a confounding bias that could have had a significant impact on the results. Third, results from this study cannot be generalized to the city of Ouagadougou, but they provide an idea of what can be expected in similar contexts.

The study also has some strengths. Specifically, this study used prospective longitudinal data covering a relatively long observation period. Our analysis method allows us to examine the relationship between health status and school trajectory in greater detail than previous studies. The study made it possible to look at various components of school trajectory—namely, age at school entry, repetition, and school dropout. Furthermore, this study addresses an important gap in the literature on the relationship between stunting and school trajectory.

Our findings imply that more effort should be made to prevent stunting; for example, raising caregivers’ awareness about child nutrition and adequate management of children’s health problems could improve outcomes. Also, those who shape the educational system—from teachers and principals to policymakers and government officials in charge of the educational system—must pay careful attention to student populations to detect and help children who are (even moderately) stunted to give them the best possible chance to pursue a successful education. Stunted children should benefit from appropriate care and learning conditions. Future research is needed to confirm our findings with a focus on rural areas. These studies could be conducted from a life-course perspective by considering entry to school, repeating a year, and dropping out globally rather than individually. They could also cover a longer observation period and consider several measures of stunting during childhood. Future research is also needed to understand the causal mechanisms by which stunting can affect school performance.

## Conclusion

The results show that schooling is affected in several ways for children who are stunted. Their school entry is more likely to be delayed and they are more likely to repeat a grade, which could lead to these children being overage for school and then having low education levels. The relationship between stunting and school dropout was inconclusive. Also, children whose mothers have no education, who live in poor households, or in informal areas tend to be older at the time they start school.

## Supporting information

S1 Checklist STROBE statement—checklist of items that should be included in reports of observational studies. (DOCX)

S1 Table Description of household hygiene score. (DOCX)

S2 Table Description of household food security score. (DOCX)

S3 Table Difference of predicted value of age at school entry by level of height-for-age over respondents’ characteristics [95% confidence interval]. (DOCX)

S4 Table Estimation of the Poisson regression model and the Poisson instrumental variable model. (DOCX)

S5 Table Comparison of baseline characteristics of participants with or without data on age at school entry. (DOCX)

S1 Fig Flow chart describing samples. (TIF)

S2 Fig Change^a^ in years of age at school entry according to a one-unit change in the initial value of the height-for-age z-score. ^**a**^Model was adjusted for sex, year of birth, month of birth, household socioeconomic status, mother’s education, place of residence.
(TIF)

S3 Fig Change^a^ in incidence of repetition according to a one-unit change in the initial value of the height-for-age z-score. ^**a**^Model was adjusted for sex, year of birth, month of birth, household socioeconomic status, mother’s education, place of residence.
(TIF)

S4 Fig Change^a^ in incidence of dropout according to a one-unit change in the initial value of the height-for-age z-score. ^**a**^Model was adjusted for sex, year of birth, month of birth, household socioeconomic status, mother’s education, place of residence.
(TIF)

## References

[1] Grantham-McGregor S, Cheung YB, Cueto S, Glewwe P, Richter L, Strupp B. Developmental potential in the first 5 years for children in developing countries. The Lancet. 2007;369(9555):60–70. doi: 10.1016/S0140-6736(07)60032-4 17208643 PMC2270351

[2] Fregonese F, Siekmans K, Kouanda S, Druetz T, Ly A, Diabaté S, et al. Impact of contaminated household environment on stunting in children aged 12–59 months in Burkina Faso. Journal of Epidemiology and Community Health. 2017;71(4):356–63. doi: 10.1136/jech-2016-207423 27986863

[3] Walker SP, Wachs TD, Meeks Gardner J, Lozoff B, Wasserman GA, Pollitt E, et al. Child development: risk factors for adverse outcomes in developing countries. The Lancet. 2007;369(9556):145–57. doi: 10.1016/S0140-6736(07)60076-2 17223478

[4] Dewey KG, Begum K. Long-term consequences of stunting in early life. Maternal & Child Nutrition. 2011;7(s3):5–18. doi: 10.1111/j.1740-8709.2011.00349.x 21929633 PMC6860846

[5] Alderman H, Behrman JR, Lavy V, Menon R. Child health and school enrollment: A longitudinal analysis. Journal of Human Resources. 2001:185–205. doi: 10.2307/3069675

[6] Glewwe P, Jacoby HG, King EM. Early childhood nutrition and academic achievement: a longitudinal analysis. Journal of Public Economics. 2001;81(3):345–68. doi: 10.1016/S0047-2727(00)00118-3

[7] Behrman JR. The Impact of Health and Nutrition on Education. The World Bank Research Observer. 1996;11(1):23–37. doi: 10.2307/3986477

[8] Wisniewski SLW. Child nutrition, health problems, and school achievement in Sri Lanka. World Development. 2010;38(3):315–32. doi: 10.1016/j.worlddev.2009.09.009

[9] Sunny BS, DeStavola B, Dube A, Kondowe S, Crampin AC, Glynn JR. Does early linear growth failure influence later school performance? A cohort study in Karonga district, northern Malawi. PLOS ONE. 2018;13(11):e0200380. doi: 10.1371/journal.pone.0200380 30395573 PMC6218019

[10] Yamauchi F. Early Childhood Nutrition, Schooling, and Sibling Inequality in a Dynamic Context: Evidence from South Africa. Economic Development and Cultural Change. 2008;56(3):657–82. doi: 10.1086/533542

[11] Gandhi M, Teivaanmaki T, Maleta K, Duan X, Ashorn P, Cheung YB. Child development at 5 years of age predicted mathematics ability and schooling outcomes in Malawian adolescents. Acta Paediatrica. 2013;102(1):58–65. doi: 10.1111/apa.12021 22957670

[12] Wolde T, Belachew T. Chronic undernutrition (stunting) is detrimental to academic performance among primary schools of adolescent children: a randomized cross sectional survey in Southern Ethiopia. BMC Res Notes. 2019;12(1):142. doi: 10.1186/s13104-019-4160-0 30876451 PMC6419846

[13] Gandhi M, Ashorn P, Maleta K, Teivaanmaki T, Duan X, Cheung YB. Height gain during early childhood is an important predictor of schooling and mathematics ability outcomes. Acta Paediatrica. 2011;100(8):1113–8. doi: 10.1111/j.1651-2227.2011.02254.x 21366692

[14] Milman A, Frongillo EA, de Onis M, Hwang J-Y. Differential Improvement among Countries in Child Stunting Is Associated with Long-Term Development and Specific Interventions. The Journal of Nutrition. 2005;135(6):1415–22. doi: 10.1093/jn/135.6.1415 15930446

[15] Walker SP, Wachs TD, Grantham-McGregor S, Black MM, Nelson CA, Huffman SL, et al. Inequality in early childhood: risk and protective factors for early child development. The Lancet. 2011;378(9799):1325–38. doi: 10.1016/S0140-6736(11)60555-2 21944375

[16] Kamano PJ, Rakotomalala R, Bernard J-M, Husson G, Reuge N. Les défis du système éducatif Burkinabè en appui à la croissance économique. Working paper. World Bank; 2010. Available from: https://dakar.iiep.unesco.org/sites/default/files/2021-09/resen_burkina.pdf.

[17] Coulidiati-Kielem J. Les facteurs déterminants de l’efficacité pédagogique des établissements secondaires: une analyse critique de l’échec scolaire au Burkina Faso. Doctorate thesis, Université de Bourgogne. 2007. Available from: https://theses.hal.science/tel-00259098/file/07087b.pdf.

[18] Direction Générale des Études et des Statistiques Sectorielles. Annuaire statistique de l’enseignement primaire 2017–2018. Ouagadougou, Burkina Faso: Ministère de l’Education Nationale et de l’Alphabétisation; 2018. Available from: http://cns.bf/IMG/pdf/annuaire_du_primaire__2017_2018-2.pdf.

[19] Ministère de la santé. Rapport de l’Enquête nutritionnelle nationale 2016. Ouagadougou, Burkina Faso: Direction de la nutrition; 2016. Available from: https://reliefweb.int/report/burkina-faso/burkina-faso-enqu-te-nutritionnelle-nationale-2016.

[20] Djourdebbé FB. Facteurs environnementaux immédiats et santé des enfants dans les zones de l’observatoire de population de Ouagadougou (Burkina Faso). Doctoral thesis, Université de Montréal. 2015. Available from: https://papyrus.bib.umontreal.ca/xmlui/handle/1866/13592.

[21] Rossier C, Soura A, Baya B, Compaoré G, Dabiré B, Dos Santos S, et al. Profile: the Ouagadougou health and demographic surveillance system. International Journal of Epidemiology. 2012;41(3):658–66. doi: 10.1093/ije/dys090 22685112 PMC3396324

[22] WHO, UNICEF. WHO child growth standards and the identification of severe acute malnutrition in infants and children: a joint statement by the World Health Organization and the United Nations Children’s Fund. Geneva, Switzerland: World Health Organization; 2009. Available from: http://www.ncbi.nlm.nih.gov/books/NBK200775. 24809116

[23] Vidmar SI, Cole TJ, Pan H. Standardizing anthropometric measures in children and adolescents with functions for egen: Update. Stata Journal. 2013;13(2):366–78.

[24] World Health Organization. WHO child growth standards: length/height-for-age, weight-for-age, weight-for-length, weight-for-height and body mass index-for-age: methods and development. World Health Organization; 2006. Available from: https://www.who.int/publications/i/item/924154693X.

[25] Rossier C, Soura A, Lankoande B. Migration et santé à la périphérie de Ouagadougou. Une première analyse exploratoire. QUETELET/QUETELET JOURNAL. 2013;1(1):91–118.

[26] Stevens GA, Finucane MM, Paciorek CJ, Flaxman SR, White RA, Donner AJ, et al. Trends in mild, moderate, and severe stunting and underweight, and progress towards MDG 1 in 141 developing countries: a systematic analysis of population representative data. Lancet. 2012;380(9844):824–34. doi: 10.1016/S0140-6736(12)60647-3 22770478 PMC3443900

[27] Allison PD. Discrete-Time Methods for the Analysis of Event Histories. Sociological Methodology. 1982;13:61–98.

[28] Allison PD. Event history and survival analysis. Second ed. Los Angeles: SAGE; 2014.

[29] Lin A, Arnold BF, Afreen S, Goto R, Huda TMN, Haque R, et al. Household environmental conditions are associated with enteropathy and impaired growth in rural Bangladesh. Am J Trop Med Hyg. 2013;89(1):130–7. doi: 10.4269/ajtmh.12-0629 23629931 PMC3748469

[30] Coates J, Swindale A, Bilinsky P. Household Food Insecurity Access Scale (HFIAS) for measurement of food access: indicator guide: version 3. Washington, D.C.: Food and Nutrition Technical Assistance Project, Academy for Educational Development; 2007. Available from: https://www.fao.org/fileadmin/user_upload/eufao-fsi4dm/doc-training/hfias.pdf.

[31] Sumsion RM, June HM, Cope MR. Measuring Food Insecurity: The Problem with Semantics. Foods. 2023;12(9):1816. doi: 10.3390/foods12091816 37174353 PMC10178861

[32] Burkina Faso. Loi N°013–2007 portant loi d’orientation de l’éducation. Ouagadougou, Burkina Faso: Assemblée Nationale; 2007.

[33] Daniels MC, Adair LS. Growth in young Filipino children predicts schooling trajectories through high school. The Journal of nutrition. 2004;134(6):1439–46. doi: 10.1093/jn/134.6.1439 15173409

[34] Santos DN, Assis AMO, Bastos ACS, Santos LM, Santos CAST, Strina A, et al. Determinants of cognitive function in childhood: A cohort study in a middle income context. BMC Public Health. 2008;8(1):202. doi: 10.1186/1471-2458-8-202 18534035 PMC2442073

[35] McDonald CM, Manji KP, Kupka R, Bellinger DC, Spiegelman D, Kisenge R, et al. Stunting and Wasting Are Associated with Poorer Psychomotor and Mental Development in HIV-Exposed Tanzanian Infants. The Journal of Nutrition. 2012;143(2):204–14. doi: 10.3945/jn.112.168682 23256148 PMC3542911

[36] Sudfeld CR, McCoy DC, Fink G, Muhihi A, Bellinger DC, Masanja H, et al. Malnutrition and Its Determinants Are Associated with Suboptimal Cognitive, Communication, and Motor Development in Tanzanian Children. The Journal of Nutrition. 2015;145(12):2705–14. doi: 10.3945/jn.115.215996 26446481

[37] Moyi P. Household characteristics and delayed school enrollment in Malawi. International Journal of Educational Development. 2010;30(3):236–42. doi: 10.1016/j.ijedudev.2009.11.008

[38] Seshie-Nasser HA, Oduro AD. Delayed primary school enrolment among boys and girls in Ghana. International Journal of Educational Development. 2016;49:107–14. doi: 10.1016/j.ijedudev.2015.12.004

[39] Gansaonré RJ, Moore L, Bleau L-P, Kobiané J-F, Haddad S. Stunting, age at school entry and academic performance in developing countries: A systematic review and meta-analysis. Acta Paediatrica. 2022;111(10):1853–61. doi: 10.1111/apa.16449 35691004

[40] Satriawan E. Essays on evaluation of the effectiveness of public health programs on child’s well-being: Evidence from Indonesia family life survey. Doctoral dissertation, Michigan State University. 2009. Available from: http://ovidsp.ovid.com/ovidweb.cgi?T=JS&PAGE=reference&D=psyc7&NEWS=N&AN=2010-99010-165.

[41] Kar BR, Rao SL, Chandramouli BA. Cognitive development in children with chronic protein energy malnutrition. Behavioral and Brain Functions. 2008;4(1):31. doi: 10.1186/1744-9081-4-31 18652660 PMC2519065

[42] Bogin B, MacVean RB. Growth status, age, and grade as predictors of school continuation for Guatemalan Indian children. Am J Phys Anthropol. 1987;73(4):507–13. doi: 10.1002/ajpa.1330730413 3661688

[43] Mendez MA, Adair LS. Severity and timing of stunting in the first two years of life affect performance on cognitive tests in late childhood. Journal of Nutrition. 1999;129(8):1555–62. doi: 10.1093/jn/129.8.1555 10419990

[44] Glewwe P, Jacoby HG. An economic analysis of delayed primary school enrollment in a low income country: the role of early childhood nutrition. The review of Economics and Statistics. 1995:156–69. doi: 10.2307/2110001

[45] Lalou R, LeGrand TK. Child mortality in the urban and rural sahel. Population: An English Selection. 1997;9:147–68.

[46] Stegelin DA. Early Literacy Education: First Steps Toward Dropout Prevention. Effective Strategies for School Improvement and Dropout Prevention. 2002.

[47] Donkor A. Parental Involvement in Education in Ghana: The Case of a Private Elementary School. International Journal about Parents in Education. 2023;4(1). doi: 10.54195/ijpe.18166

[48] Paul R, Rashmi R, Srivastava S. Does lack of parental involvement affect school dropout among Indian adolescents? evidence from a panel study. PLOS ONE. 2021;16(5):e0251520. doi: 10.1371/journal.pone.0251520 33970973 PMC8109829

