# Supplementary material for: Stunting and academic trajectory in urban settings of Burkina Faso
Source: PLoS One. 2024 Dec 11;19(12):e0314051. doi: 10.1371/journal.pone.0314051 (PMC11633982; doi:10.1371/journal.pone.0314051)
Supplement: S1 Checklist — (DOCX) [file pone.0314051.s001.docx]

S1 STROBE checklist : STROBE Statement—checklist of items that should be included in reports of observational studies

|  | Item No. | Recommendation | Page  No. | Relevant text from manuscript |
| --- | --- | --- | --- | --- |
| **Title and abstract** | 1 | (*a*) Indicate the study’s design with a commonly used term in the title or the abstract | 2 | Lines 28-29: We used longitudinal data from the Ouagadougou Health and Demographic Surveillance System |
|  |  | (*b*) Provide in the abstract an informative and balanced summary of what was done and what was found | 2-3 | Lines 20-46; Abstract |
| Introduction | | | |  |
| Background/rationale | 2 | Explain the scientific background and rationale for the investigation being reported | 4-5 | Lines 49-80; Introduction |
| Objectives | 3 | State specific objectives, including any prespecified hypotheses | 5 | Lines 76-80; The primary objective of this research was to estimate the age at which children start school according to levels of height-for-age z-score (stunting). A secondary objective was to estimate the gain in terms of age at school entry that would be associated with an improvement in child height-for-age z-score. Our third objective was to explore the relationship between stunting, grade repetition, and school dropout. |
| Methods | | | |  |
| Study design | 4 | Present key elements of study design early in the paper | 6 | Lines 96-100; To assess the relationship between child health and schooling, we used longitudinal data collected from children living in the five city districts covered by the Ouagadougou Health and Demographic Surveillance System (OHDSS). Cross-sectional health data collected in 2010, when children were under 5 years old, were combined with their subsequent longitudinal schooling data. |
| Setting | 5 | Describe the setting, locations, and relevant dates, including periods of recruitment, exposure, follow-up, and data collection | 6 | Lines 100-110; Study design and setting |
| Participants | 6 | (*a*) *Cohort study*—Give the eligibility criteria, and the sources and methods of selection of participants. Describe methods of follow-up  *Case-control study*—Give the eligibility criteria, and the sources and methods of case ascertainment and control selection. Give the rationale for the choice of cases and controls  *Cross-sectional study*—Give the eligibility criteria, and the sources and methods of selection of participants | 6 | Lines 112-113; Our sample consisted of children who participated in the health survey and who had an educational follow-up. |
|  |  | (*b*) *Cohort study*—For matched studies, give matching criteria and number of exposed and unexposed  *Case-control study*—For matched studies, give matching criteria and the number of controls per case | N/A | This is not a matched study |
| Variables | 7 | Clearly define all outcomes, exposures, predictors, potential confounders, and effect modifiers. Give diagnostic criteria, if applicable | 8-10 | Lines 145-157; Outcome measures.  Lines 158-169; Main independent variable.  Lines 170-199; Potential confounders or modifiers. |
| Data sources/ measurement | 8* | For each variable of interest, give sources of data and details of methods of assessment (measurement). Describe comparability of assessment methods if there is more than one group | 7-8 | Lines 112-125; Data sources |
| Bias | 9 | Describe any efforts to address potential sources of bias | 11-12 | Lines 223-237; Sensitivity analysis |
| Study size | 10 | Explain how the study size was arrived at | 7-8 | Lines 131-144, Study population |

Continued on next page

| Quantitative variables | 11 | Explain how quantitative variables were handled in the analyses. If applicable, describe which groupings were chosen and why | 10 | Lines 202-203; The linearity assumption not being respected; we included the height-for-age z-score as a quadratic and cubic term. |
| --- | --- | --- | --- | --- |
| Statistical methods | 12 | (*a*) Describe all statistical methods, including those used to control for confounding | 10 to 11 | Lines 200-222; Statistical analysis |
|  |  | (*b*) Describe any methods used to examine subgroups and interactions | 11 | Lines 213-215; Statistical analysis |
|  |  | (*c*) Explain how missing data were addressed | N/A | Missing data were on dependent variable. See lines 118-125. The they exclude from analysis.  See also lines 402-411. |
|  |  | (*d*) *Cohort study*—If applicable, explain how loss to follow-up was addressed  *Case-control study*—If applicable, explain how matching of cases and controls was addressed  *Cross-sectional study*—If applicable, describe analytical methods taking account of sampling strategy | 10 to 11 | Lines 216-222; Statistical analysis. |
|  |  | (*e*) Describe any sensitivity analyses | 11 to 12 | Lines 223-237; Sensitivity analysis |
| Results | | | | |
| Participants | 13* | (a) Report numbers of individuals at each stage of study—eg numbers potentially eligible, examined for eligibility, confirmed eligible, included in the study, completing follow-up, and analysed | 6 to 7 and 14 | Lines 115-130; Study population. Table 1. Age at school entry, incidence of grade repetition, incidence of dropout, and baseline characteristics of participants. |
|  |  | (b) Give reasons for non-participation at each stage | N/A |  |
|  |  | (c) Consider use of a flow diagram | N/A |  |
| Descriptive data | 14* | (a) Give characteristics of study participants (eg demographic, clinical, social) and information on exposures and potential confounders | 14 | Table 1. Age at school entry, incidence of grade repetition, incidence of dropout, and baseline characteristics of participants. |
|  |  | (b) Indicate number of participants with missing data for each variable of interest | 14 | Table 1. Age at school entry, incidence of grade repetition, incidence of dropout, and baseline characteristics of participants. |
|  |  | (c) *Cohort study*—Summarise follow-up time (eg, average and total amount) | N/A |  |
| Outcome data | 15* | *Cohort study*—Report numbers of outcome events or summary measures over time | 13 and 14 | Lines 249-251. Table 1. Age at school entry, incidence of grade repetition, incidence of dropout, and baseline characteristics of participants. |
|  |  | *Case-control study—*Report numbers in each exposure category, or summary measures of exposure | N/A | This is not a case-control study |
|  |  | *Cross-sectional study—*Report numbers of outcome events or summary measures | N/A | This is not a cross-sectional study |
| Main results | 16 | (*a*) Give unadjusted estimates and, if applicable, confounder-adjusted estimates and their precision (eg, 95% confidence interval). Make clear which confounders were adjusted for and why they were included | 13 to 18 | Lines 249-251. Table 1. Age at school entry, incidence of grade repetition, incidence of dropout, and baseline characteristics of participants.  Lines 259-318. Fig. 1. Predicted value of age at school entry (in years), by level of height-for-age. Table 2. Change in age at school entry [95% confidence interval]. Fig 2. Predicted value of age at school entry by height-for-age z-score and respondents’ characteristics. Fig 3. Predicted incidence of repetition and incidence of dropout by level of height-for-age. Table 3. Changea in incidence of repetition [95% confidence interval]. |
|  |  | (*b*) Report category boundaries when continuous variables were categorized | N/A | There was no categorization of continuous variables |
|  |  | (*c*) If relevant, consider translating estimates of relative risk into absolute risk for a meaningful time period | N/A | There was no estimation of relative risk |

Continued on next page

| Other analyses | 17 | Report other analyses done—eg analyses of subgroups and interactions, and sensitivity analyses | 18 | Lines 319-324; Sensitivity analysis |
| --- | --- | --- | --- | --- |
| Discussion | | | | |
| Key results | 18 | Summarise key results with reference to study objectives | 18 to 22 | Lines 325-401; Discussion |
| Limitations | 19 | Discuss limitations of the study, taking into account sources of potential bias or imprecision. Discuss both direction and magnitude of any potential bias | 22 | Lines 402-417; Discussion |
| Interpretation | 20 | Give a cautious overall interpretation of results considering objectives, limitations, multiplicity of analyses, results from similar studies, and other relevant evidence | N/A |  |
| Generalisability | 21 | Discuss the generalisability (external validity) of the study results | 22 | Lines 415-417; Results from this study cannot be generalized to cities like Ouagadougou, but they provide an idea of what can be expected in this type of context. |
| Other information | |  | | |
| Funding | 22 | Give the source of funding and the role of the funders for the present study and, if applicable, for the original study on which the present article is based | N/A | Submitted along with the online application form |

*Give information separately for cases and controls in case-control studies and, if applicable, for exposed and unexposed groups in cohort and cross-sectional studies.

**Note:** An Explanation and Elaboration article discusses each checklist item and gives methodological background and published examples of transparent reporting. The STROBE checklist is best used in conjunction with this article (freely available on the Web sites of PLoS Medicine at http://www.plosmedicine.org/, Annals of Internal Medicine at http://www.annals.org/, and Epidemiology at http://www.epidem.com/). Information on the STROBE Initiative is available at www.strobe-statement.org.
