# Supplementary material for: Stunting and academic trajectory in urban settings of Burkina Faso
Source: PLoS One. 2024 Dec 11;19(12):e0314051. doi: 10.1371/journal.pone.0314051 (PMC11633982; doi:10.1371/journal.pone.0314051)
Supplement: S1 Table — (DOCX) [file pone.0314051.s002.docx]

| S1 Table. Description of household hygiene score | |  |
| --- | --- | --- |
| Household hygiene | N (758) | % |
| Type of toilet |  |  |
| Nature | 30 | 4.0 |
| Simple latrine | 688 | 90.7 |
| Improved latrine | 40 | 5.3 |
|  |  |  |
| Toilet restricted or shared |  |  |
| Outside courtyard | 75 | 9.9 |
| Shared with courtyard members | 155 | 20.5 |
| restricted to household members | 528 | 69.6 |
|  |  |  |
| Main source of drinking water in the last 7 days | |  |
| Well, rain, water sellers | 25 | 3.3 |
| Public pump | 84 | 11.1 |
| Public fountain | 526 | 69.4 |
| Tap on yard | 123 | 16.2 |
|  |  |  |
| Floor material |  |  |
| Land | 705 | 93.1 |
| Cement, paving stone | 52 | 6.9 |
|  |  |  |
| Household hygiene score^†^ | 758 | 6 (5;7) |
| ^†^Median(q1;q3). |  |  |
