# Supplementary material for: Stunting and academic trajectory in urban settings of Burkina Faso
Source: PLoS One. 2024 Dec 11;19(12):e0314051. doi: 10.1371/journal.pone.0314051 (PMC11633982; doi:10.1371/journal.pone.0314051)
Supplement: S2 Table — (DOCX) [file pone.0314051.s003.docx]

| S2 Table. Description of household food security score. | | |  |
| --- | --- | --- | --- |
| Household food security | | N (758) | % |
| Concerned about lack of food in the last 30 days | |  |  |
|  | Never | 185 | 24.4 |
|  | Rarely | 175 | 23.1 |
|  | Sometimes | 280 | 36.9 |
|  | Often | 118 | 15.6 |
| Not eating favorite foods for lack of means | |  |  |
|  | Never | 185 | 24.4 |
|  | Rarely | 239 | 31.5 |
|  | Sometimes | 272 | 35.9 |
|  | Often | 62 | 8.2 |
| Forced to eat the same food for lack of means | |  |  |
|  | Never | 183 | 24.1 |
|  | Rarely | 184 | 24.3 |
|  | Sometimes | 269 | 35.5 |
|  | Often | 122 | 16.1 |
| Forced to eat unwanted food for lack of means | |  |  |
|  | Never | 227 | 30.0 |
|  | Rarely | 257 | 33.9 |
|  | Sometimes | 220 | 29.0 |
|  | Often | 54 | 7.1 |
| Reduce the amount eaten due to lack of food | |  |  |
|  | Never | 216 | 28.5 |
|  | Rarely | 273 | 36.0 |
|  | Sometimes | 225 | 29.7 |
|  | Often | 44 | 5.8 |
| Reduce the number of meals due to lack of food | |  |  |
|  | Never | 257 | 33.9 |
|  | Rarely | 251 | 33.1 |
|  | Sometimes | 206 | 27.2 |
|  | Often | 44 | 5.8 |
| Going to bed hungry for lack of food |  |  |  |
|  | Never | 319 | 42.1 |
|  | Rarely | 207 | 27.3 |
|  | Sometimes | 201 | 26.5 |
|  | Often | 31 | 4.1 |
| Nothing to eat in the house for lack of means | |  |  |
|  | Never | 439 | 57.9 |
|  | Rarely | 161 | 21.2 |
|  | Sometimes | 124 | 16.4 |
|  | Often | 34 | 4.5 |
| Spend a day without eating for lack of means | |  |  |
|  | Never | 553 | 73.0 |
|  | Rarely | 145 | 19.1 |
|  | Sometimes | 52 | 6.9 |
|  | Often | 8 | 1.0 |
|  |  |  |  |
| Food security score^†^ |  | 758 | 18 (13;23) |
| ^†^Median(q1-q3). |  |  |  |
