# Supplementary material for: Stunting and academic trajectory in urban settings of Burkina Faso
Source: PLoS One. 2024 Dec 11;19(12):e0314051. doi: 10.1371/journal.pone.0314051 (PMC11633982; doi:10.1371/journal.pone.0314051)
Supplement: S4 Table — (DOCX) [file pone.0314051.s005.docx]

| S4 Table. Estimation of the Poisson regression model and the Poisson instrumental variable model. | | | | | | | | |
| --- | --- | --- | --- | --- | --- | --- | --- | --- |
| Variables | | Poisson model | | |  | PIV model | | |
|  |  | IRR | 95% CI | |  | IRR | 95% CI | |
| Height-for-age z-score | | 0.95 | [0.94; | 0.96] |  | 0.94 | [0.90; | 0.98] |
|  |  |  |  |  |  |  |  |  |
| Sex |  |  |  |  |  |  |  |  |
|  | Girl (Ref.) |  |  |  |  |  |  |  |
|  | Boy | 0.98 | [0.96; | 1.00] |  | 0.98 | [0.95; | 1.00] |
| Month of birth | |  |  |  |  |  |  |  |
|  | January-August (Ref.) |  |  |  |  |  |  |  |
|  | September-December | 0.96 | [0.93; | 0.98] |  | 0.96 | [0.93; | 0.98] |
| Year of birth | |  |  |  |  |  |  |  |
|  | 2005 (Ref.) |  |  |  |  |  |  |  |
|  | 2006 | 1.01 | [0.97; | 1.06] |  | 1.01 | [0.96; | 1.07] |
|  | 2007 | 0.92 | [0.88; | 0.97] |  | 0.93 | [0.88; | 0.97] |
|  | 2008 | 0.83 | [0.79; | 0.88] |  | 0.83 | [0.78; | 0.88] |
|  | 2009 | 0.81 | [0.76; | 0.86] |  | 0.80 | [0.74; | 0.86] |
| Household Socioeconomic status^β^ | | |  |  |  |  |  |  |
|  | Poor (Ref.) |  |  |  |  |  |  |  |
|  | Non-poor | 0.89 | [0.87; | 0.91] |  | 0.89 | [0.87; | 0.91] |
| Mother education^γ^ | |  |  |  |  |  |  |  |
|  | No education (Ref.) |  |  |  |  |  |  |  |
|  | Some education | 0.98 | [0.95; | 1.00] |  | 0.98 | [0.95; | 1.01] |
| Place of residence^δ^ | |  |  |  |  |  |  |  |
|  | Formal area (Ref.) |  |  |  |  |  |  |  |
|  | Informal area | 1.08 | [1.05; | 1.11] |  | 1.07 | [1.04; | 1.11] |
| Rho statistic | |  |  |  |  | 0.02 | [-0.03; | 0.06] |
| PIV: Poisson Instrumental Variable; 95% CI (Confidence interval); IRR: Incidence Rate Ratio.  ^β^ The less-poor households were grouped together with the rich and intermediate households. They were grouped together because of the low number of rich households.  ^γ^ Mothers with no education were those with no formal education. Those who attended to formal education (elementary education or more) were grouped into “some education.”  ^δ^ Informal area comprised Polesgo, Nonghin, and Nioko II. They are devoid of zoning plan and basic public services (schools, health centers, etc.). Formal neighborhoods (Kilwin and Tanghin) are well structured and provided with public infrastructure.  Model was adjusted for sex, year of birth, month of birth, household socioeconomic status, mother’s education, place of residence. | | | | | | | | |
