# Supplementary figures and images for: Stunting and academic trajectory in urban settings of Burkina Faso
Source: PLoS One. 2024 Dec 11;19(12):e0314051. doi: 10.1371/journal.pone.0314051 (PMC11633982; doi:10.1371/journal.pone.0314051)

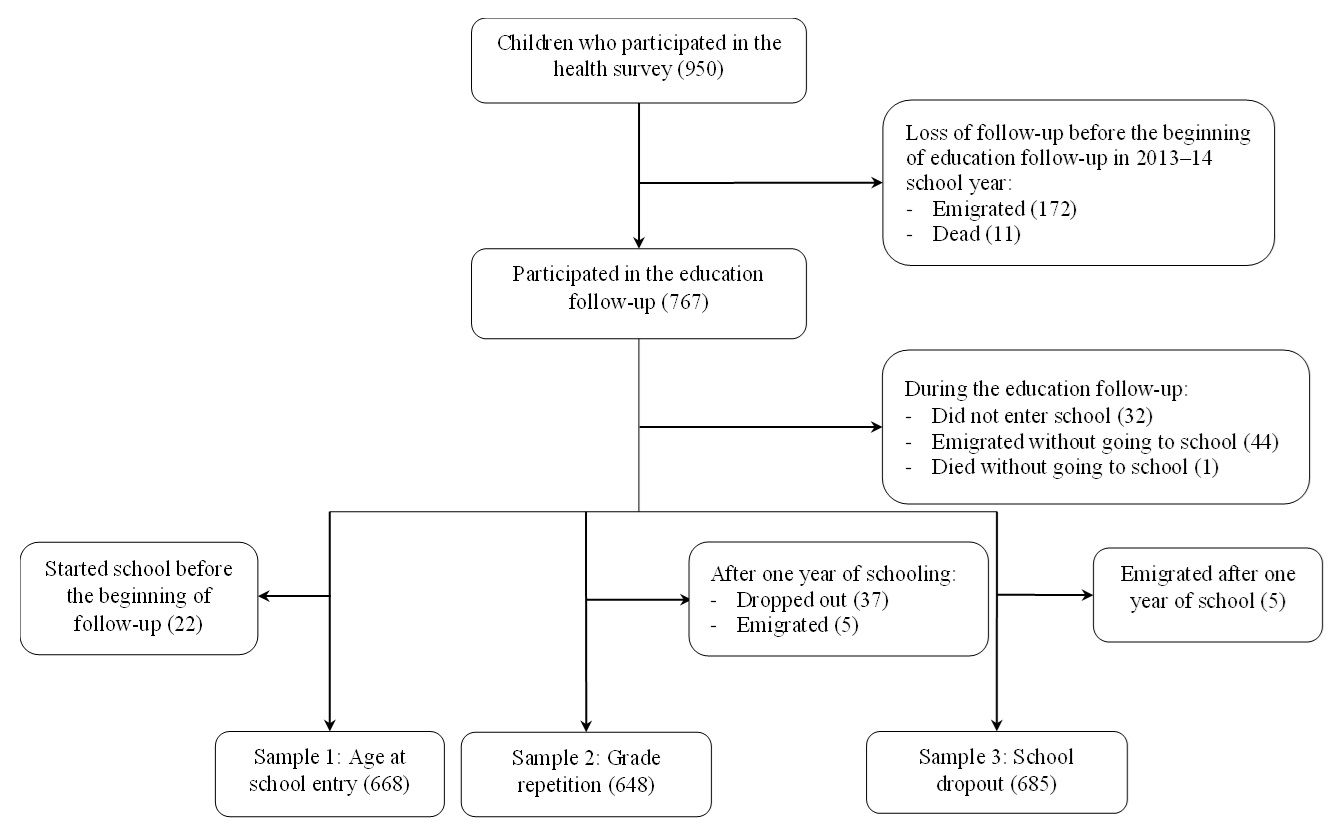

Supplement: S1 Fig — (TIF) [file pone.0314051.s007.tif]

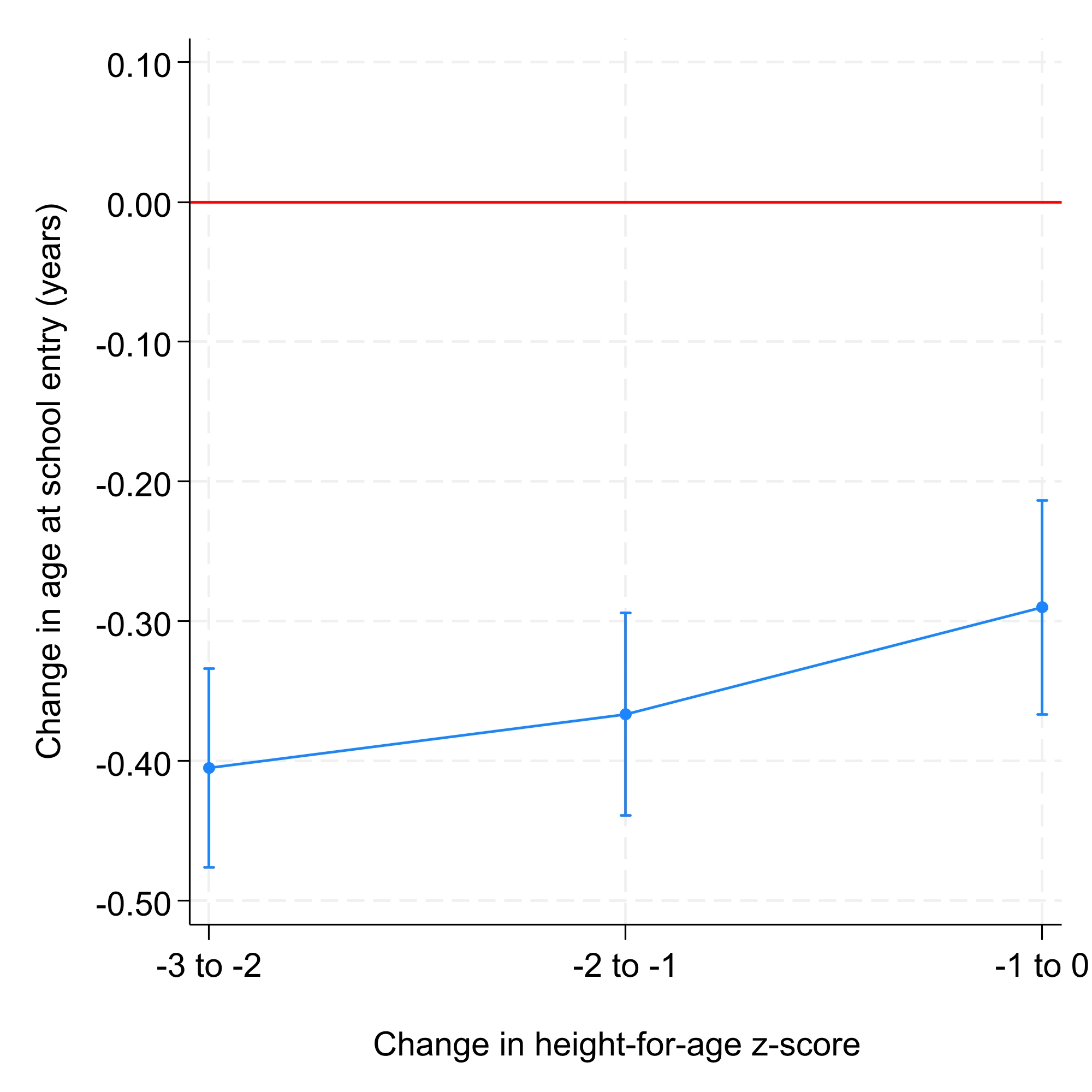

Supplement: S2 Fig — aModel was adjusted for sex, year of birth, month of birth, household socioeconomic status, mother’s education, place of residence. (TIF) [file pone.0314051.s008.tif]

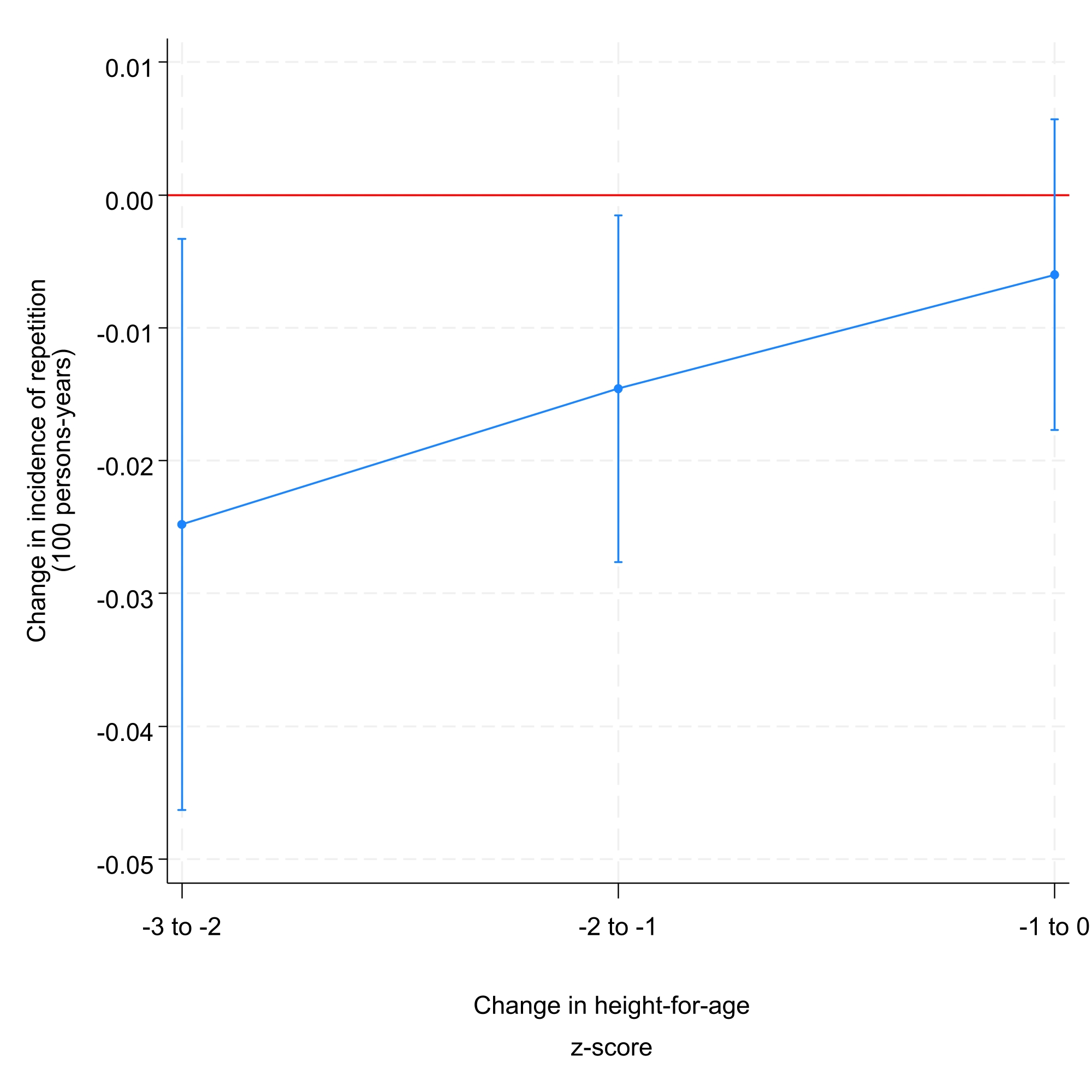

Supplement: S3 Fig — aModel was adjusted for sex, year of birth, month of birth, household socioeconomic status, mother’s education, place of residence. (TIF) [file pone.0314051.s009.tif]

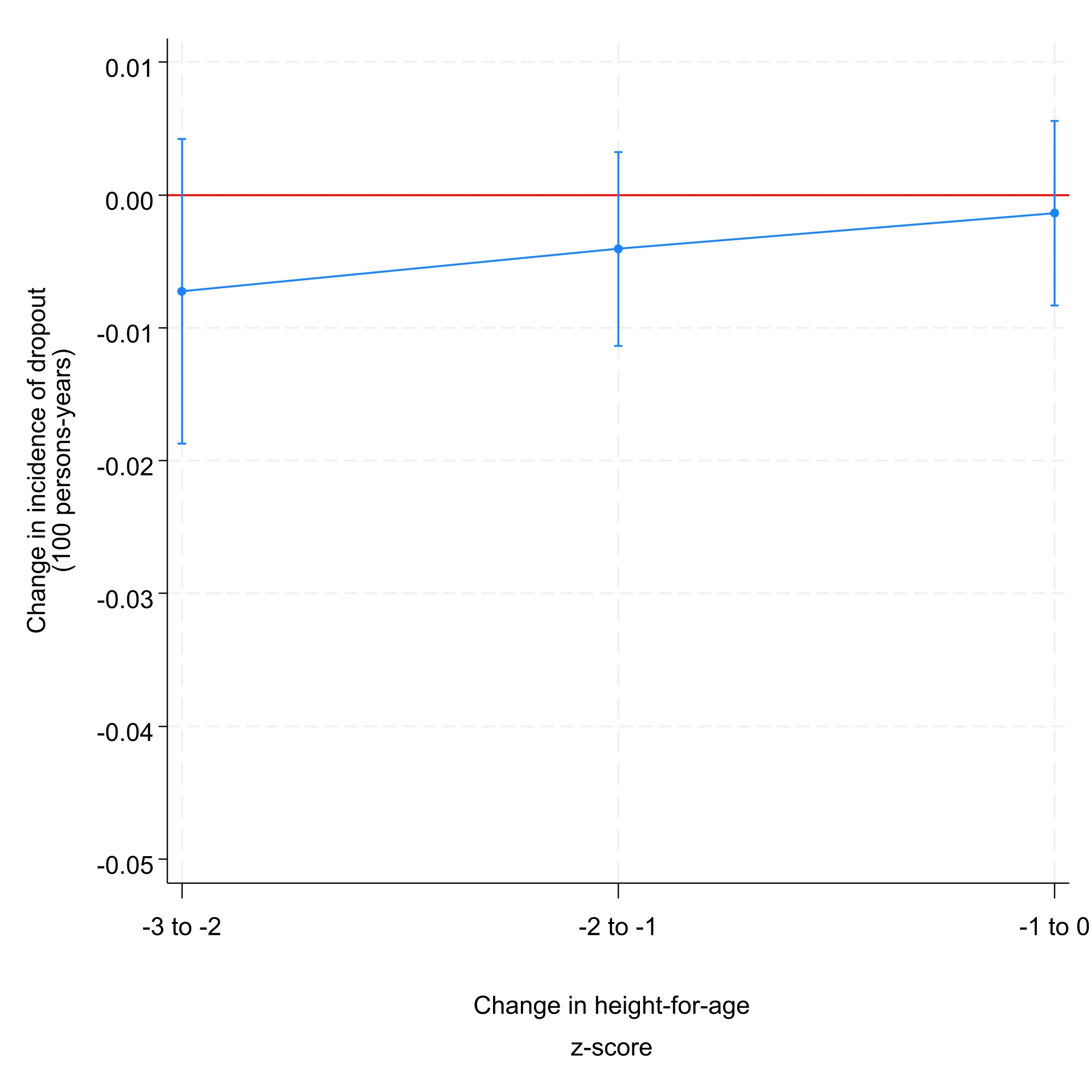

Supplement: S4 Fig — aModel was adjusted for sex, year of birth, month of birth, household socioeconomic status, mother’s education, place of residence. (TIF) [file pone.0314051.s010.tif]
